# Electrophysiological Evaluation of Paralytic Shellfish Toxins in Bivalves

**DOI:** 10.3390/md24100365

**Published:** 2026-10-19

**Authors:** Runa Yonekura, Satoshi Numano, Ryuichi Watanabe, Ikkei Yoshida, Fumiko Obara, Junko Okumura, Yuko Cho, Yuta Kudo, Kazuo Nagasawa, Mari Yotsu-Yamashita, Keiichi Konoki

**Affiliations:** 1 Graduate School of Agricultural Science, Tohoku University, 468-1 Aramaki-Aza-Aoba, Aoba-ku, Sendai 980-8572, Japan; yonekura.runa.r8@dc.tohoku.ac.jp (R.Y.); yuko.cho.a4@tohoku.ac.jp (Y.C.); yuta.kudo.d5@tohoku.ac.jp (Y.K.); mari.yamashita.c1@tohoku.ac.jp (M.Y.-Y.); 2 Fisheries Technology Institute, Japan Fisheries Research and Education Agency, Yokohama, Kanagawa 236-8648, Japan; numano_satoshi55@fra.go.jp; 3 Japan Food Inspection Corporation, Sapporo Inspection Office, Rabo Bldg., Kita 1-jo Nishi 21-3-17, Chuo-ku, Sapporo, Hokkaido 064-0821, Japan; i_yoshida@jffic.or.jp (I.Y.); f_obara@jffic.or.jp (F.O.); j_okumura@jffic.or.jp (J.O.); 4 Frontier Research Institute for Interdisciplinary Sciences, Tohoku University, 6-3 Aramaki-Aza-Aoba, Aoba-ku, Sendai 980-8578, Japan; 5 Department of Biotechnology and Life Science, Tokyo University of Agriculture and Technology, Koganei, Tokyo 184-8588, Japan; fx7239@go.tuat.ac.jp

**Keywords:** paralytic shellfish toxins, saxitoxin, electrophysiology, LC-MS/MS, bivalve mollusks

## Abstract

Paralytic shellfish toxins (PSTs) represented by saxitoxin (STX) are lethal; pre-shipment toxicity testing of shellfish is mandatory. In Japan, screening methods that reliably guarantee toxin concentrations below shipment regulatory limits (0.8 mg STX·2HCl eq./kg) are permitted. In this study, we aimed to develop an electrophysiological method for PSTs by utilizing their property of inhibiting voltage-gated sodium channels (Na_v_) expressed in mouse neuroblastoma Neuro 2a cells. In addition to the three reference standards of dcSTX, GTX2/3, and C1/2, extracts from the digestive glands of toxic *Patinopecten yessoensis* and those from the whole edible parts of *P. yessoensis*, *Mimachlamys nobilis*, *Perna viridis*, and *Mytilus galloprovincialis* were tested. As a result, the toxicity required to inhibit Na_v_ by 50% was 1–4 µg STX·2HCl eq./kg, which was well below the shipment regulatory limits and roughly consistent across the three reference standards and all the bivalve extracts. A calibration curve was constructed by globally fitting the dose–response curves obtained from electrophysiological experiments using various bivalve extracts. The electrophysiological toxicity of each extract was then estimated using this calibration curve. The correlation coefficient (r) between the electrophysiological toxicity and the toxicity determined by LC-MS/MS was 0.90. Thus, these findings provide fundamental insights for establishing an electrophysiological screening method for PSTs.

## 1. Introduction

**Figure 1:**
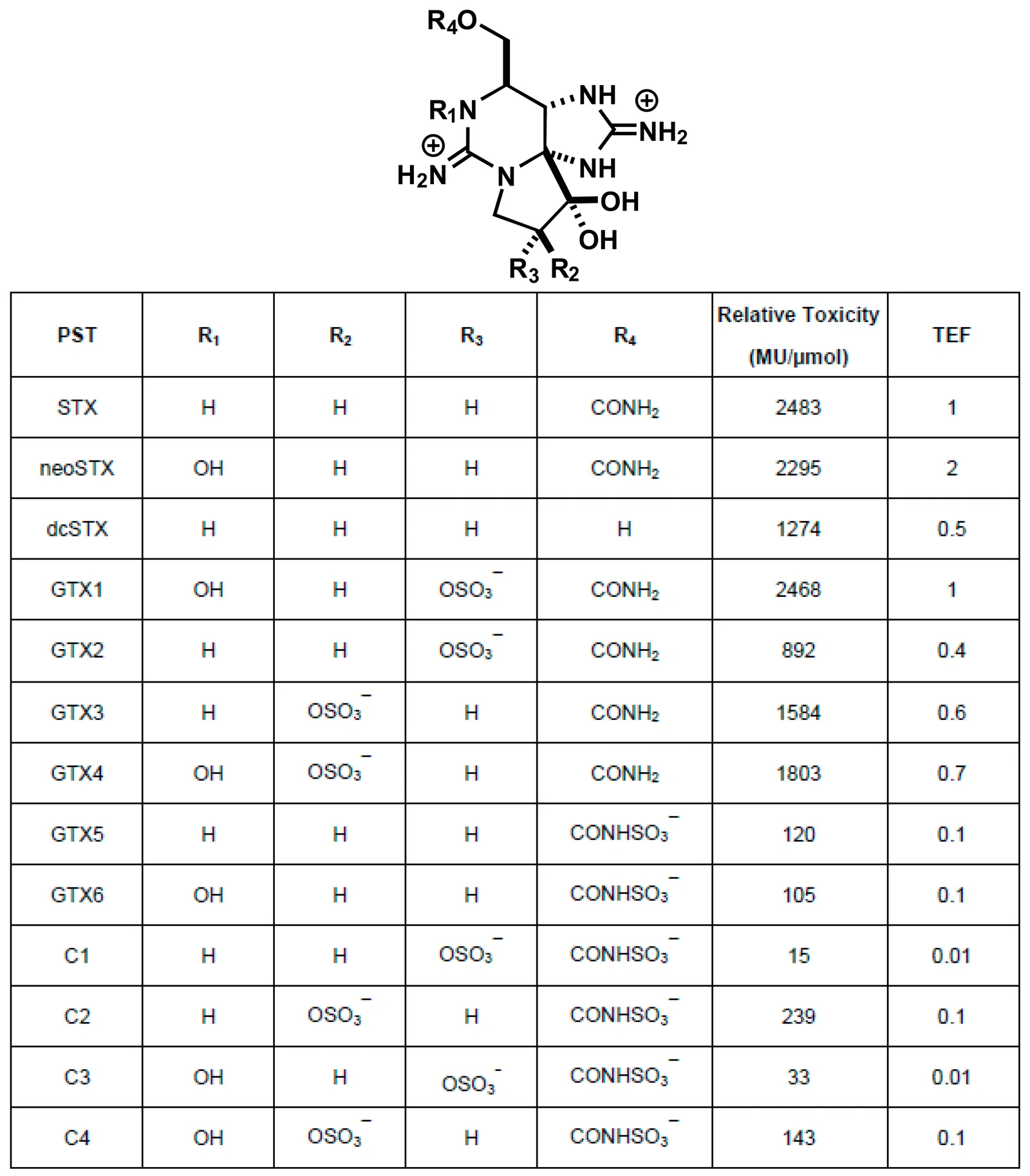
Structures of paralytic shellfish toxins (PSTs), their toxicities in mice, and toxicity equivalent factors (TEFs) [7,8,9].

Paralytic shellfish toxins (PSTs) is a collective term for a group of compounds comprising more than 50 analogs (Figure 1), with saxitoxin (STX) [1,2] being the most well-known among them [3]. PSTs are produced by dinoflagellates of the genera *Alexandrium* and *Gymnodinium catenatum*, and are known to accumulate in bivalves, such as scallops. Consumption of such contaminated bivalves can cause paralysis and, in the worst cases, even death [4,5]. Therefore, pre-shipment toxicity testing is conducted worldwide [6]. Bivalves with PST levels exceeding legal limits (4 MU/g, equally 0.8 mg STX·2HCl eq./kg) are subject to shipping restrictions in Japan, and these restrictions have ensured that there have been no reported fatalities associated with bivalves distributed in the market since 1980.

For a long time, the mouse bioassay (MBA), an intraperitoneal injection method in mice, was used as the standard for pre-shipment toxicity testing. Toxicity was determined according to Sommer’s table, with 1 MU defined as the toxicity level that caused the death of a mouse weighing 20 g within 15 min; individual bivalves with a toxicity level of 4 MU or more per gram of edible portion were prohibited from shipment [1]. Over time, challenges with respect to accuracy and sensitivity of the MBA were recognized, and awareness of animal welfare also simultaneously increased, leading to a transition to instrumental analytical methods of testing, resulting in the development of spectroscopic methods using fluorescent dyes [10], fluorescent HPLC [7] and LC/MS methods [11,12]. None of these methods directly measures toxicity. Instead, they calculated the total toxicity by multiplying the determined amount of each component by a toxicity equivalency factor (TEF, assigned when saxitoxin’s toxicity is defined as 1; Figure 1) and summing the toxicities of each component. Shipment is restricted if the total toxicity exceeds 0.8 mg STX·2HCl eq./kg [13]. These methods have been used in the United States, Europe, Canada, Australia, and New Zealand. Following the CODEX standards established by the FAO and WHO [9], Japan enacted a law in 2015 recommending instrumental analysis for the detection of shellfish toxins, not limited to PSTs, and has fully transitioned to the instrumental analysis of diarrhetic shellfish toxins. As mentioned earlier, PSTs comprise a cocktail of over 50 structurally similar compounds [3]; however, a complete transition to a mass spectrometry–based method has not yet been realized owing to several challenges. These include the fact that only about a dozen reference standards are available, the near-total absence of reference standards for M toxins [14,15,16]—a newly recognized subgroup—and, particularly in Japan, the inability to use STX owing to restrictions under the Act on the Prohibition of Chemical Weapons [17]. An ELISA method using antibodies was also developed [18,19,20,21]. Unlike HPLC or LC-MS/MS methods, the ELISA method measures the total PST content, as done by the MBA. However, the ELISA method was also faced with challenges because each compound had varying affinity for the antibody, resulting in an inaccurate estimation of the total amount of the substance and, consequently, the overall toxicity of the substance not being appropriately reflected. Therefore, the ELISA method needs to be improved by preparing a reference standard mixture tailored to seasonal variations in composition and enhancing affinity of the compounds for the antibody through prior chemical modification.

PSTs bind to voltage-gated sodium channels (Na_v_) with high affinity and specificity, blocking the flow of sodium ions [22]. Na_v_s are molecular targets for various neurotoxins, which have been used as research tools to elucidate the structure and function of Na_v_s. There are presumably five main neurotoxin-binding sites in Na_v_, and PSTs bind to “Site 1,” the same site where tetrodotoxin (TTX)—derived from pufferfish—and μ-conotoxin—derived from cone snails—bind. With advances in cryo-EM single-particle analysis technology, the structures of membrane proteins have been rapidly elucidated. The three-dimensional structure of human Na_v_ was determined for the first time in 2019 [23], revealing that STX and TTX bound near the entrance that controls ion selectivity [24], which largely confirmed the structure of the complex previously inferred from pharmacological and electrophysiological experiments using radioligands and recombinant Na_v_ mutants [25,26,27].

Electrophysiological methods are the most sensitive techniques for detecting Na_v_ and have been used to elucidate the mechanism of action of PSTs. Vélez et al. [28] performed electrophysiological evaluations of Na_v_1.4 sensitivity to extracts obtained from PST-contaminated mussels and rock oysters, demonstrating a high correlation between the STX equivalents determined using MBA and those determined using electrophysiological experiments. Furthermore, Alonso et al. [29] used an automated electrophysiological system capable of performing multiple measurements to simultaneously evaluate the sensitivity of Na_v_1.1–Na_v_1.7 to nine PST reference standards approved by the National Research Council of Canada. Based on the structure–activity relationships between PSTs and Na_v_ subtypes, Alonso et al. [29] identified Na_v_1.2 and Na_v_1.6 as subtypes that correlated with the results of MBA.

Previously, we had investigated the subtype selectivity of TTX analogues and chemically synthesized STX derivatives using electrophysiological methods [30,31,32]. During the screening phase, we used mouse neuroblastoma Neuro 2a (N2a) cells, which express endogenous Na_v_. Based on this experience, we believed that PST-mediated Na_v_ inhibitory activity could be readily detected even at levels well below the legal limit for shipping (4 MU/g in MBA) and detection limit (0.875 MU/g in MBA) [1]. In this study, we measured the Na_v_ inhibitory activity in N2a cells using three PST reference standards—dcSTX, GTX2/3, and C1/2—and extracts from PST-contaminated bivalves and digestive glands of the scallop *Patinopecten yessoensis*, whose compositional profiles had also been determined via LC-MS/MS [32]. These results demonstrate that electrophysiological methods can be used to quantify PST toxicity.

## 2. Results

### 2.1. Evaluation Using PSTs Reference Standards

**Figure 2:**
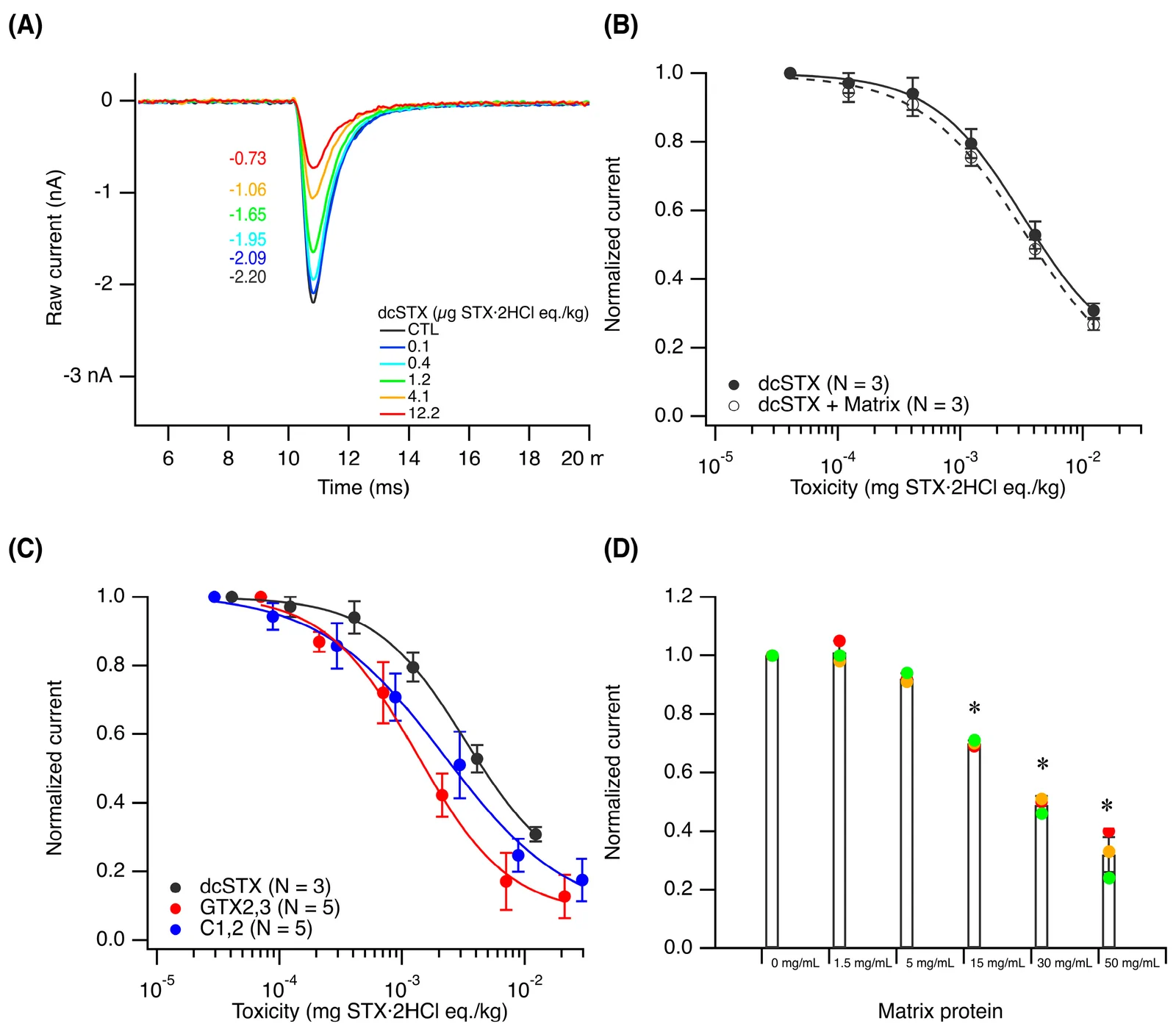
Dose-dependent inhibition of endogenous Na_v_ in N2a cells by dcSTX. (**A**) Raw data of depolarization-evoked sodium current in the presence of 0.1, 0.4, 1.2, 4.1, and 12.2 µg STX·2HCl eq./kg of dcSTX. (**B**) Dose–response curves for dcSTX and a non-toxic scallop extract spiked with dcSTX (4 MU/g). (**C**) Dose–response curves for the reference standards (see Table S2). Tukey’s test showed significant differences between dcSTX and C1/2 (*p* = 0.0084) and between dcSTX and GTX2/3 (*p* = 0.00039). (**D**) An extract prepared from a non-toxic scallop was diluted 10-fold (50 mg-protein/mL), 16-fold (30 mg-protein /mL), 32-fold (15 mg-protein /mL), 100-fold (5 mg-protein/mL), and 320-fold (1.5 mg-protein/mL) in extracellular buffer for electrophysiological experiments to perfuse N2a cells and record Na_v_ (N = 3). The bars and error bars represent the means and SDs, respectively, while the colored dots indicate individual measurements (N = 3). One-way ANOVA followed by Tukey’s test showed significant differences from the control only at 15, 30, and 50 mg-protein/mL (*p* < 0.05), as indicated by asterisks.

We used whole-cell patch-clamp techniques to study endogenously expressed Na_v_ in N2a cells. First, the noise level was measured in a whole-cell configuration at a holding potential of −100 mV and was found to be 32.7 ± 12.7 pA (N = 11). The magnitude was unrelated to the magnitude of the sharp downward current observed when the cell was depolarized to −10 mV. Upon depolarization to −10 mV, Na_v_ opens and closes within just 2–3 ms, allowing sodium ions to flow from the extracellular space into the intracellular space, thereby generating a sharp peak current (Figure 2A). Based on the methods described by Oshima et al. [7,8], the concentrations of the PST reference standards—dcSTX, GTX2/3, and C1/2—were converted to toxicity units, and extracellular solutions containing each of these compounds at low to high toxicity levels were prepared and used to perfuse N2a cells. As a result, extracellular solutions containing 0, 0.1, 0.4, 1.2, 4.1, and 12.2 µg STX·2HCl eq./kg dcSTX reduced peak currents in a toxicity-dependent manner (Figure 2A). When normalized current values were plotted against toxicity and the data were fitted to the Hill equation, a downward-sloping sigmoid curve was obtained. The IC_50_, defined as the concentration that reduces the current by half, was calculated to be 4.13 ± 0.45 µg STX·2HCl eq./kg (Mean ± SD, Figure 2B). Similarly, the IC_50_ value of GTX2/3 and C1/2 was calculated to be 1.41 ± 0.36 (N = 5) and 2.38 ± 0.87 µg STX·2HCl eq./kg (N = 5), respectively (Mean ± SD, Figure 2C and Table S2). One-way ANOVA revealed a significant difference among the IC_50_ values of dcSTX, C1/2, and GTX2/3 (*p* = 0.00053). Tukey’s multiple-comparison test showed significant differences between dcSTX and C1/2 (*p* = 0.0084) and between dcSTX and GTX2/3 (*p* = 0.00039), whereas no statistically significant difference was observed between C1/2 and GTX2/3 (*p* = 0.085).

### 2.2. Pilot Study Using Digestive Glands of Scallops

Electrophysiological evaluation of PSTs in the digestive glands is particularly important. In scallops, PSTs accumulate primarily in the digestive glands. The shipment of scallop adductor muscles is permitted in some prefectures of Japan even during the shipping restriction period if the adductor muscles are removed at a government-designated processing facility. The PST composition in four digestive glands, Samples 1–4, from the scallops was analyzed using LC-MS/MS [32] (Figure S1A), and the total toxicity was calculated by multiplying the amount of each component by its toxicity equivalency factor, dilution factor, and molecular weight of STX·2HCl and summing them. As a result, the total toxicity of Samples 1–4 was found to be 2.43, 5.62, 7.99, and 4.28 mg STX·2HCl eq./kg, respectively. N2a cells were perfused with a diluted extract prepared from the digestive glands of scallops, and a dose-dependent inhibition of Na_v_ was observed (Figure S1B). The IC_50_ value of Samples 1–4, calculated from the fitted downward-sloping sigmoid curves, was 1.14 ± 0.41, 1.86 ± 0.76, 2.10 ± 0.57, and 1.70 ± 0.21 µg STX·2HCl eq./kg, respectively (Table S2). One-way ANOVA (*p* = 0.038) followed by Tukey’s multiple-comparison test was performed to compare the IC_50_ values among the samples. A significant difference was observed only between Samples 1 and 3 (*p* = 0.037). Although the intrinsic total toxicity varied among Samples 1–4, their IC_50_ values were generally comparable, with only a minor but statistically significant difference between Samples 1 and 3, and were close to those of the reference standards GTX2/3 and C1/2 [33]. 

### 2.3. Pilot Study Using the Entire Edible Portion of Various Bivalves

**Figure 3:**
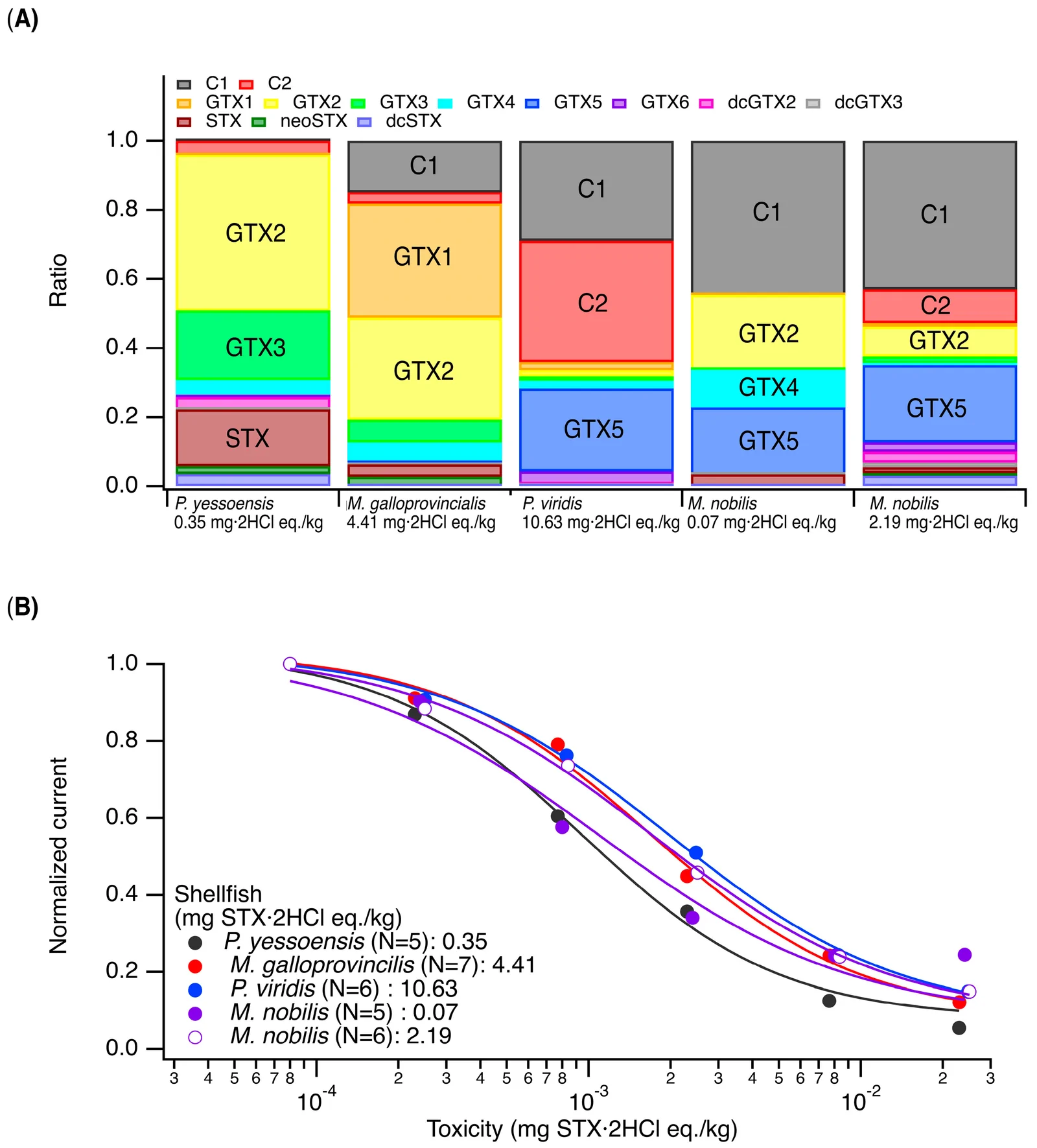
Dose-dependent inhibition of endogenous Na_v_ in N2a cells by toxic whole-organ extract of toxic shellfish. (**A**) The PST composition from *Patinopecten yessoensis* (0.35 mg STX·2HCl eq./kg), *Mytilus galloprovincialis* (4.41 mg STX·2HCl eq./kg), *Perna viridis* (10.63 mg STX·2HCl eq./kg), and *Mimachlamys nobilis* (0.07 and 2.19 mg STX·2HCl eq./kg) was determined using LC-MS/MS. We aimed to quantify the 15 components of the PSTs and TTX for which reference standards were available (Table S1), detected 13 of them, and converted the molar ratios so that their sum equaled 1. (**B**) Dose–response curves for the scallop extracts. Normalized Na_v_ currents were plotted against toxicity and fitted to the Hill equation to determine IC_50_ values (Table S2).

In actual pre-shipment toxicity testing, the entire edible portion of various types of bivalves is subject to testing because the PST composition can vary depending on the harvest time and location. Therefore, we selected *Patinopecten yessoensis*, *Mimachlamys nobilis*, *Perna viridis,* and *Mytilus galloprovincialis* and investigated their toxin composition using LC-MS/MS (Figure 3A). The major components of *P. yessoensis* were found to be GTX2/3 and STX; those of *M. galloprovincialis* were C1 and GTX1/2; and those of *P. viridis* were C1/2 and GTX5. Among the two *M. nobilis* specimens, one had C1 and GTX2/4/5 as its major components, whereas the other had C1/2 and GTX2/5 as its major components [33]. The total toxicity levels were 0.35 (*P. yessoensis*), 4.41 (*M. galloprovincialis*), 10.63 (*P. viridis*), and 0.07 and 2.19 (*M. nobilis*) mg STX·2HCl eq./kg. Electrophysiological experiments revealed that the IC_50_ values were 1.43 ± 0.02 (N = 5), 1.76 ± 0.29 (N = 7), 1.80 ± 0.45 (N = 6), 1.25 ± 0.42 (N = 5), and 2.31 ± 1.06 µg STX·2HCl eq./kg (N = 6), respectively (Figure 3B and Table S2). One-way ANOVA revealed no significant difference among the five groups (*p* = 0.075).

### 2.4. Experiment to Verify the Matrix Effect

The samples analyzed in this study were prepared by adding an equal volume of acetic acid solution to finely chopped digestive glands, heating the mixture to extract the contents, and centrifuging it to obtain the supernatant, which was then subjected to whole-cell patch-clamp recordings. The PSTs assay using HPLC [7] or LC-MS/MS [11,12] requires further purification and stipulates the use of samples that have undergone solid-phase extraction and ultrafiltration of this supernatant; therefore, the test solution used in the electrophysiological experiments in this study may be subject to matrix effects. Therefore, it was necessary to verify the lack of matrix effects for the test solutions that had not undergone solid-phase extraction. To 0.31 mL of 10 µM dcSTX, 1.69 mL of the non-toxic scallop extract was added to prepare 2 mL of a mixture containing 2 MU/g (292 µg STX·2HCl eq./kg). The solution was then diluted with the extracellular buffer for the whole-cell patch-clamp experiments to five different concentrations—0, 0.1, 0.4, 1.2, 4.1, and 12.2 µg STX·2HCl eq./kg—to perfuse N2a cells (Figure 2B). At the highest toxicity of 12.2 µg STX·2HCl eq./kg, the ratio of the non-toxic scallop extract to the extracellular buffer was approximately 1:16. As a result, the IC_50_ was calculated to be 3.34 ± 0.35 µg STX·2HCl eq./kg, and no statistically significant difference was observed between dcSTX alone and dcSTX in the non-toxic scallop extract (one-way ANOVA, *p* = 0.075). To confirm these findings, we diluted an extract from the non-toxic scallop extract with the extracellular solution for electrophysiological experiments by 10-fold (50 mg-protein/mL), 16-fold (30 mg-protein /mL), 32-fold (15 mg-protein /mL), 100-fold (5 mg-protein/mL), and 320-fold (1.5 mg-protein/mL) in extracellular buffer for electrophysiological experiments to perfuse N2a cells and record Na_v_ (N = 3) (Figure 2D). The observed current values at each matrix concentration were normalized. One-way ANOVA followed by Tukey’s multiple-comparison test was used to compare the groups. A significant difference from the control was observed at 15, 30 and 50 mg/mL (*p* < 0.05). But no Na_v_-inhibitory activity was observed at 100-fold or higher dilutions.

## 3. Discussion

In electrophysiological experiments, although the measurement system is housed inside a Faraday cage, there is still noise that cannot be ignored. This noise level can be reduced primarily by periodic recoating of the anode and cathode. The noise level at a holding potential of −100 mV in a whole-cell configuration was 32.7 ± 12.7 pA (N = 11). If the detection limit is set to s/n > 3, the detection limit can be approximately 100 pA, and if the quantification limit is set to s/n > 10, the quantification limit can be approximately 330 pA. In practice, as the peak Na_v_ current value for each cell is generally in the range of 1–3 nA, it is difficult to clearly define the detection limit or quantification limit when the current values are normalized. If the magnitude of the Na_v_ current is 1 nA, normalizing 330 pA yields 0.33, which means that an inhibition rate of 67% or lower is considered a valid quantitative value; if the maximum current is 3 nA, normalizing 330 pA yields 0.11, which means that an inhibition rate of 89% or lower is considered a valid quantitative value. This noise level should also apply to the low inhibition rate range. Therefore, when the maximum current is 1 nA, the current values that satisfy the s/n > 3 criterion are valid within the 33–67% inhibition rate range; similarly, when the maximum current is 3 nA, they are valid within the 11–89% inhibition rate range. The range of inhibition rates that satisfy the detection limit is surprisingly narrow, especially when the peak Na_v_ current is small; ideally, observations should be made at an inhibition rate of 50%; however, predicting IC_50_ is difficult. Therefore, it is necessary to set several dilutions of a PST-positive extract, one of which may result in an approximately 50% inhibition and determine the toxicity by referring to the calibration curve, which could be a dose–response curve of the PST reference standards.

The IC_50_ should also be determined for the following reason. Demonstrating a correlation between Na_v_-inhibitory activity and toxicity in mice is a prerequisite for testing PSTs using electrophysiological methods [28]. In this study, we evaluated this hypothesis by estimating IC_50_ values from dose–response curves of normalized currents plotted against toxicity. The results indicated that the IC_50_ values were generally comparable despite differences in the original toxicities of the samples, although statistically significant differences were observed between some groups. When the three reference standards were tested, the IC_50_ values for GTX2/3 and C1/2 were comparable, with no statistically significant difference between them. In contrast, the IC_50_ value for dcSTX was significantly higher than those for GTX2/3 and C1/2 (*p* < 0.05). GTX2/3 and C1/2 are reference standards that were purchased from the National Research Council of Canada and stored under appropriate conditions. Synthetic dcSTX [34] was quantified by LC-MS using its enantiomer, the concentrations of which were determined using LC-NMR [32]. An earlier study that examined different Na_v_ subtypes in N2a cells using RT-PCR reported that Na_v_1.7 is the major Na_v_ subtype expressed in N2a cells, with Na_v_1.2, 1.3, and 1.4 also being expressed [35]. The IC_50_ value for Na_v_1.7 was the lowest for dcSTX (89 nM), followed by GTX2/3 (1500 nM) and C1/2 (3700 nM), indicating that dcSTX can be a more potent inhibitor by an order of magnitude [29]. Therefore, we concluded that this does not explain why dcSTX has a higher IC_50_ value than the other two compounds. Although N2a cells express multiple Na_v_ isoforms, the qualitative RT-PCR data available in the literature do not allow us to determine the relative expression levels or functional contribution of each Na_v_ isoform. Differences in Na_v_ isoform composition may be one possible explanation, but this possibility remains to be experimentally validated.

**Figure 4:**
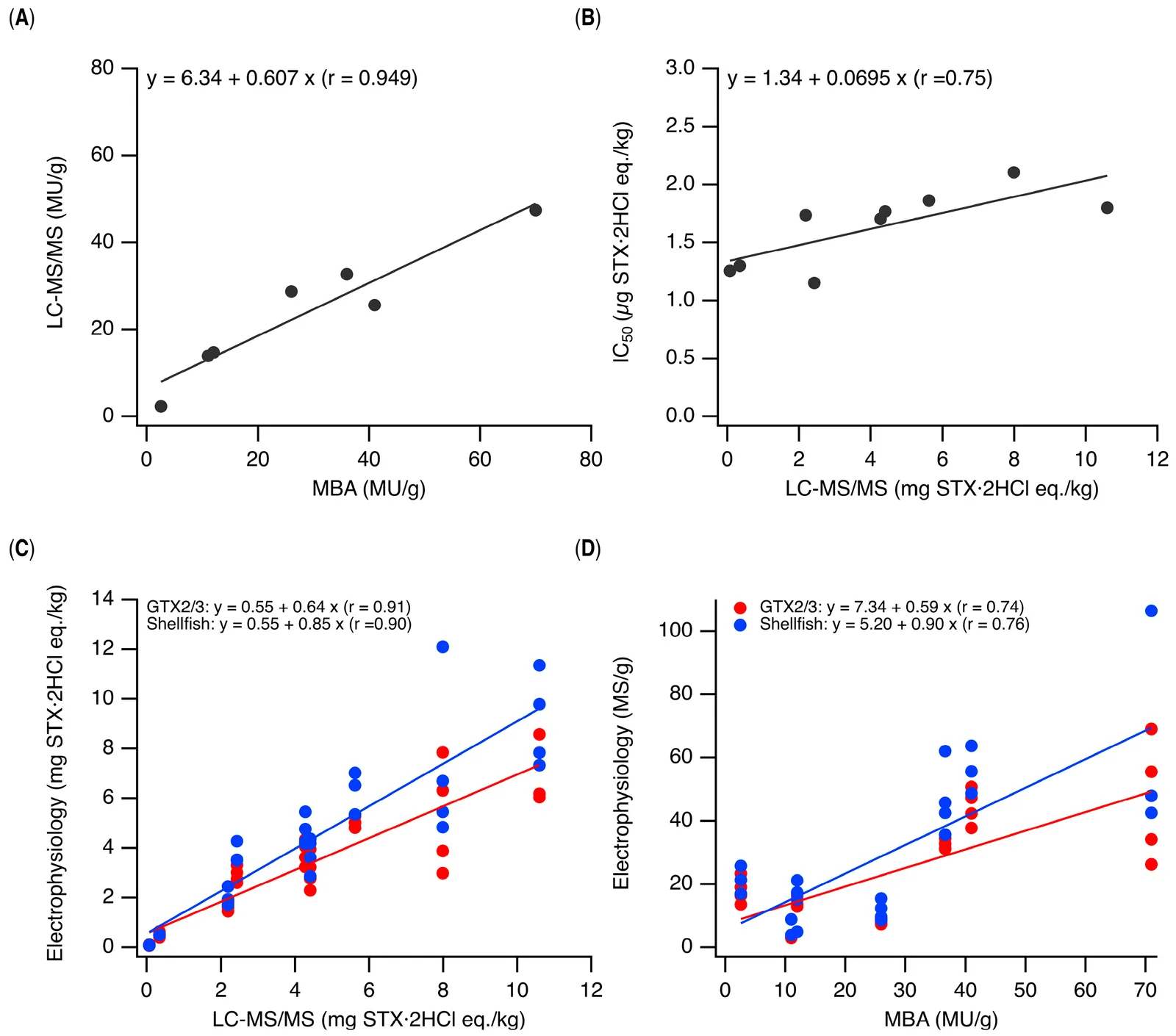
Comparison of PST toxicity determined by LC-MS/MS, mouse bioassay, and electrophysiological methods. (**A**) Correlation between toxicity values assessed using MBA and LC-MS/MS. The results of MBA and LC-MS/MS analyses using extracts from four scallop digestive glands and three edible parts of bivalves are plotted (See Table S2). (**B**) The IC_50_ values listed in Table S2 were plotted against toxicity determined using LC-MS/MS, and linear regression analysis was performed. (**C**) Correlation between toxicity measured using LC-MS/MS and that measured via electrophysiological experiments. Using the dose–response curve for GTX2/3 or the averaged dose–response curve from all the PST-contaminated shellfish extracts as calibration curves (Table S3), we reevaluated the toxicity of PST-contaminated extracts and plotted the results against the toxicity measured using LC-MS/MS. Linear regression analysis was performed, and the results are shown along with the correlation coefficient (inserted in the upper left corner). (**D**) Correlation between electrophysiologically determined toxicity and that determined via MBA (See Table S2). Using dose–response curves of extracts from GTX2/3 or total PST-contaminated samples as calibration curves, we reevaluated the toxicity of PST-contaminated extracts and plotted the results against the toxicity determined by MBA. Linear regression analysis was performed, and the results, along with the correlation coefficient (inserted in the upper left corner).

On the other hand, despite differences in the toxin composition of PST-contaminated bivalve extracts, no statistically significant differences in IC_50_ values were observed among the four bivalve species in the electrophysiological experiments (*p* > 0.05), with IC_50_ values ranging approximately from 1 to 2 µg STX·2HCl eq./kg. (Figure 3 and Table S2). When data obtained from the digestive glands of scallops and various bivalves were plotted, with toxicity determined by LC-MS/MS on the x-axis and IC_50_ values on the y-axis, there was a very insignificant change in IC_50_ values with increasing toxicity (Figure 4B). These results generally support the hypothesis that the toxicity required to inhibit Na_v_ currents by 50% is comparable across different bivalve samples, regardless of their inherent toxicity, although a statistically significant difference in IC_50_ values was observed between two scallop digestive gland samples (Samples 1 and 3; *p* = 0.037, Figure S1 and Table S2). It was also found that PST-positive samples and GTX2/3 or C1/2 showed nearly complete overlap. This may be expected because the PST-positive samples used in this study consisted primarily of GTX2/3 and C1/2, and because other components, such as STX, dcSTX, and NeoSTX, even when present, exhibit Na_v_-inhibitory activity and mouse toxicity levels that are similar to those of GTX2/3 and C1/2.

However, it must be noted that the fitted line in Figure 4B exhibits a slightly positive slope. When diluting bivalve extracts with different toxicities to the same level of toxicity for N2a cell perfusion, the dilution factor for the less toxic bivalve extract is inevitably lower, which we believe makes it more prone to yielding a matrix effect. It is known that compounds, such as fatty acids, contained in the matrix inhibit Na_v_. However, verification using a non-toxic scallop extract revealed that the matrix effect can be eliminated if the original extract is diluted more than 100-fold for N2a perfusion (Figure 2D). As the IC_50_ for Na_v_-inhibitory activity is approximately 1–2 µg STX·2HCl eq./kg, it can be inferred that extracts with toxicity of 100–200 µg STX·2HCl eq./kg or higher are not subject to matrix effects and exhibit effects attributable to PSTs. As the bivalve extract used in this study had a minimum toxicity of 73.9 µg STX·2HCl eq./kg for *M. nobilis* (Figure 3 and Table S2), a matrix effect may be present at the higher end of its toxicity. We believe this is why the regression line shown in Figure 4B exhibits a slightly positive slope. However, the present dataset was insufficient to define a reliable toxicity threshold above which matrix interference becomes negligible. Further analysis using a larger number of samples, particularly those with low-to-intermediate toxicity, will be required to establish such a threshold.

The electrophysiological method is significantly more sensitive than MBA [28,36,37], which, according to Food Sanitation Guidelines, cannot detect toxicity levels below 175 µg STX·2HCl eq./kg (0.875 MU/g) [17]. Even if the matrix effect is considered, the fact that we were able to measure such low toxicity levels—to the point where it can be considered virtually non-toxic and far below the shipment regulatory limits (4 MU/g, equally 0.8 mg STX·2HCl eq.kg)—should be highlighted as a key finding of this study. Furthermore, another important advantage of the electrophysiological method is that it dispels concerns regarding compositional differences, which is an advantage over existing methods that require pretreatment of reference standards and solid-phase extraction of PST-contaminated extracts. Alonso et al. used a multi-sample automated electrophysiological testing system to investigate the sensitivity of each Na_v_ subtype to PST reference standards and reported that Na_v_ 1.2 and 1.6 were the subtypes most strongly correlated with mouse toxicity [29]. As mentioned earlier, Na_v_1.6 is not the major Na_v_ subtype expressed in N2a cells [35]. Each PST exhibits different dissociation constants for different Na_v_ subtypes, which are expressed at varying levels in N2a cells, which would explain why the electrophysiological method for quantifying PST using N2a cells yielded satisfactory results.

Interestingly, data obtained from both the digestive gland (Figure S1) and edible portion of scallops (Figure 3) yielded similar IC_50_ values. Initially, it was assumed that PSTs are concentrated in the digestive gland. However, the similar IC_50_ values for digestive glands and edible portions, noted in this study, can be explained by the fact that PSTs accumulate in other organs as well [38], and by the existence of M toxins [14,15,16]—a new subgroup of PSTs for which no reference standards are available —making it difficult to measure the total accumulated toxicity using conventional methods. While performing the MBA, the number of mice used must be kept to a minimum from an animal welfare perspective, making it impossible to validate the results using reference standards. Electrophysiological methods do not only ensure sensitivity and accuracy but also allow validation using reference standards; therefore, the electrophysiological methods outperform the MBA. One limitation of the present method is that, for samples with unknown toxicity and no prior LC-MS/MS data, the appropriate dilution range cannot be determined in advance. Therefore, stepwise serial dilution is necessary to identify a concentration range that produces measurable Na_v_ inhibition while maintaining sufficient current amplitude for reliable measurement. This additional step may increase the time and effort required for analysis, particularly when screening large numbers of samples.

This study aimed to develop a methodology for determining whether scallops conform to shipping guidelines using electrophysiological techniques. Therefore, using the dose–response curves for GTX2/3 as calibration curves, it is possible to calculate mouse toxicity values for appropriately diluted PST-positive samples. First, we set the dose–response curves for GTX2/3 (Figure 2C)—which had undergone global fitting and averaging—as standard curves and developed a calculation formula to convert Na_v_ current values into electrophysiological toxicity (Table S3). Using this formula, we determined the electrophysiological toxicity of each PST-contaminated extract and plotted it against the toxicity determined using LC-MS/MS (Figure 4C). Linear analysis of the plot (Figure 4C, red) revealed that the slope of the regression line was 0.64, indicating a discrepancy of approximately 36% between electrophysiological toxicity and toxicity determined using LC-MS/MS. Therefore, we applied global fitting to the dose–response data from the 11 PST-positive samples (Figure 3 and Figure S1), averaged the resulting dose–response curves, and used the averaged curve as a common calibration curve for estimating shellfish toxicity (Table S3). When this was reapplied to each PST-positive sample and the resulting electrophysiological toxicity was plotted against the toxicity determined using LC-MS/MS (Figure 4C, blue), the slope of the line improved from 0.64 to 0.85; However, the correlation coefficient remained nearly unchanged (r = 0.91 and 0.90, respectively), and the difference between the electrophysiological toxicity and the toxicity determined using LC-MS/MS decreased to 15%. As MBA remains the mainstream method in Japan, we investigated the correlation between electrophysiologically determined toxicity and toxicity determined via the MBA (Figure 4D). When dose-dependent curves for GTX2/3 and bivalve extracts were used as calibration curves, the correlation coefficients between toxicity determined via MBA and that determined electrophysiologically were 0.74 and 0.76, respectively, and the slopes were 0.59 and 0.90, respectively. The correlation between MBA and LC-MS/MS (Figure 4A) was better than that between MBA and electrophysiological methods. In MBA, PSTs as well as various other substances cause lethal toxicity in mice. As the LC-MS/MS method requires reference standards for quantification, it cannot cover all PSTs. Therefore, no single method, including electrophysiological methods, is ideal. However, we are unable to determine why the estimated toxicity measured using our electrophysiological method was lower than that measured using the other two methods.

The discrepancy may also be partly attributable to the curve-fitting procedure used to estimate electrophysiological toxicity. In the present study, concentration–response data were fitted to a four-parameter Hill equation, and global fitting with two shared parameters improved the regression and correlation with LC-MS/MS measurements to some extent. Nevertheless, the estimated IC_50_ remains dependent on the fitting of multiple parameters and may therefore be affected by variability in the concentration–response data. Accurate measurements around the midpoint of the concentration–response curve are particularly important for robust IC_50_ estimation. Further optimization of the concentration range and fitting procedure may therefore improve the quantitative accuracy of the electrophysiological assay. To estimate the toxicity of PSTs in bivalve extracts of various compositions, it is necessary to use a mixture of reference standards with appropriate compositions. Otherwise, the use of a multi-sample electrophysiological system that allows for the analysis of standard samples and various bivalve extracts on the same day using cells from the same lot is recommended, whereas the current approach uses a manually operated electrophysiological device. 

We also expect that the use of automated multi-sample electrophysiological testing systems, which have been widely adopted in recent years [29,37], particularly by pharmaceutical companies, will lower technical barriers and shorten the time from sample collection to shipment. Recently, there has been an increasing number of reports of TTX—which binds to the same site as STX and specifically inhibits Na_v_—being found in bivalves [39]; however, Réverté et al. very recently analyzed bivalve extracts containing both STX analogs and TTX using an automated electrophysiological system. Using the dose–response curves obtained with STX and TTX as calibration curves, they estimated the PST toxicity of 80 different bivalve extracts. The slope of the line obtained by plotting the electrophysiological toxicity values against the toxicity values quantified using LC-MS/MS was 0.992, with a correlation coefficient of 0.999 [40]. These results demonstrate the high reproducibility and quantitative performance achievable with automated electrophysiological systems and highlight the limitations of the manual whole-cell patch-clamp approach used in the present study, particularly with respect to throughput and variability among individual recordings. The ability to simultaneously quantify PSTs and TTXs is an advantage over ELISA, and the ability to analyze a large number of bivalve extracts simultaneously is superior to ELISA. However, one limitation of the study by Réverté et al. was that the detailed toxin composition was not reported. Independent of their study, we analyzed the same N2a cells using a manual electrophysiological apparatus in the present study. We also intended to detect TTX via our LC-MS/MS analysis (Table S1); however, it was not detected. We have previously analyzed TTX analogues in electrophysiological experiments and, therefore, would have been able to detect them as part of the total toxin content, similar to what was done by Réverté et al. Unlike Réverté et al., we used the dose–response curves of GTX2/3 and those from 11 PST-contaminated shellfish extracts as calibration curves to estimate the electrophysiological toxicity of the PST-contaminated shellfish extracts. As mentioned earlier, the electrophysiological toxicity was strongly correlated with the toxicity determined by LC-MS/MS (r = 0.91), and the slope of the regression line was 0.85, relatively close to the ideal value of 1. Nevertheless, further improvements may be necessary to enhance the quantitative accuracy of the electrophysiological method.

Taken together, the present study demonstrates the feasibility of whole-cell electrophysiology using N2a cells as a sensitive functional approach for evaluating PST toxicity in bivalve extracts. The method detected Na_v_-inhibitory activity at toxicity levels considerably below the current regulatory limit for shellfish shipment. However, the remaining differences between electrophysiological toxicity estimates and those obtained by LC-MS/MS, together with the limitations associated with manual electrophysiological measurements and the relatively small number of samples, indicate that further optimization and validation are necessary before this method can be applied to routine regulatory testing.

## 4. Materials and Methods

### 4.1. Materials

The certified reference materials GTX2/3 (lot number: 20170419) and C1/2 (lot number: 20201208) were purchased from the National Research Council of Canada (NRCC, Halifax, NS, Canada). GTX2 and GTX3 [41,42], as well as C1 and C2 [43], are equilibrium mixtures, which differ in terms of the proton configurations connected to C11. The concentrations of GTX2 and GTX3 are 102.6 and 43.5 µM, respectively, whereas those of C1 and C2 are 94.4 and 26.8 µM, respectively. The synthetic reference standard dcSTX [34,44] was calibrated using its reference standard prepared by Oshima et al. [7,32]. N2a cells were provided by the RIKEN BioResource Center (Wako, Saitama, Japan). All other chemicals were purchased from Nacalai Tesque, Inc. (Kyoto, Kyoto, Japan).

### 4.2. Specimens

Overall, 20 g of each specimen was finely ground in a food processor. The sample was then mixed with 20 mL of 1% acetic acid solution to be 500 mg/mL and boiled for 5 min. After cooling the mixture on ice, it was vortexed followed by centrifugation at 4000× *g* and 4 °C for 10 min. A part of the resulting supernatant was directly used for electrophysiological experiments. To investigate the matrix effect of the extract from a non-toxic scallop in electrophysiological experiments, the 500 mg/mL suspension obtained at this stage was diluted 10-fold, 16-fold, 32-fold, 100-fold, and 320-fold with extracellular solution prepared for electrophysiological experiments, and the resulting concentrations of 50, 30, 15, 5, and 1.5 mg-protein/mL, respectively, were used. From the remaining supernatant, 3 mL was loaded onto a pre-equilibrated Sep-Pak C18 column (1.0 g, Waters Corporation, Milford, MA, USA); the first 1.5 mL was discarded, and the subsequent 0.5 mL was collected, which was subjected to ultrafiltration (MWCO: 10 kDa), and the resulting flow-through was used for LC-MS/MS.

### 4.3. Mouse Bioassays

The mouse bioassay was performed as part of statutory pre-shipment shellfish toxicity testing at the Sapporo Inspection Office of the Japan Food Inspection Corporation, in accordance with its standard operating procedure C12000-1.

The extracts containing toxins were prepared according to the Standard Methods of Analysis in Food Safety Regulation in Japan. The sample was weighed, and an equal volume of 0.1 M HCl was added; the mixture was then finely ground using a mixer. After adjusting the pH to 3.0 ± 0.5, the mixture was boiled for 5 min. The pH was checked, and the mixture was allowed to cool. The supernatant was then collected and used for the assay. The assay was conducted via intraperitoneal administration of 1 ml to 5 ddY male mice (weighing 19–21 g) at a specialized facility licensed to perform animal experiments. The median death time was defined as the time of death of the third mouse; this median value was then referenced against Sommer’s table to determine the mouse toxicity value.

### 4.4. LC-MS/MS 

LC-MS/MS was performed as previously described [16,32]. Briefly, MS or LC-MS/MS spectra were recorded using a 4500 Qtrap mass spectrometer (SCIEX, Marlborough, MA, USA) connected to an LC-20 UHPLC System (Shimadzu Corporation, Kyoto, Kyoto, Japan). The mass spectrometer was equipped with an ESI source and set up as follows: positive ionization mode; ion spray voltage: 5500 V; ion source temperature: 200 °C curtain gas: 20 psi; analysis mode: selected reaction monitoring. An Acquity UPLC BEH amide column (Waters, 2.1 × 150 mm, 1.7 µm) was used. The mobile phase A was 0.015% formic acid containing 0.06 % ammonium hydroxide, and the mobile phase B was 70% acetonitrile/0.01% formic acid. Gradient elution was performed as follows: 0–5 min, the A:B ratio was constant at 2:98; between 5 and 7.5 min, a linear gradient was applied from 2:98 to 50:50, and this condition was maintained until 10.5 min; between 10.5 and 11 min, a linear gradient was applied from 50:50 to 2:98, and this condition was maintained until 15 min for re-equilibration. The flow rate was 0.4 mL/min. The conditions for analyzing the 14 PST reference standards and TTX on selected reaction monitoring (SRM) mode are shown in Table S1.

### 4.5. Cell Culture

N2a cells were cultured in Minimum Eagle’s Essential Medium with Earle’s salts and containing 10% (*v*/*v*) fetal bovine serum, 10 units/mL penicillin, and 10 μg/mL streptomycin (Nacalai Tesque, Inc.) at 37 °C in a humidified environment with a 5% CO_2_/air atmosphere. N2a cells, which were subcultured on a large scale and cryopreserved using Cell Banker 1 (Nippon Zenyaku Kogyo Co., Ltd., Koriyama, Fukushima, Japan), were thawed for subsequent experiments. Every 3–4 days, cells were detached and harvested using 0.25% trypsin/1 mM EDTA (Nacalai Tesque, Inc.), then diluted and seeded in a single-cell state for electrophysiological experiments. Cells diluted and seeded at a ratio of 1:8 to 1:2 were used for continuous maintenance, which was counted as one passage. The cells were maintained up to approximately the 12^th^ passage, and cells from the third passage onwards were used for electrophysiological experiments.

### 4.6. Electrophysiological Experiments

Electrophysiological experiments were performed using a whole-cell patch-clamp recording setup [30,31,32]. The extracellular buffer comprised 140 mM NaCl, 1.0 mM MgCl_2_·6 H_2_O, 5.0 mM CsCl, 1.8 mM CaCl_2_, and 10 mM HEPES (pH 7.4, adjusted with NaOH), and the osmotic pressure was adjusted to 280 mmol/kg using a Vapro Vapor Pressure Osmometer 5600C (ELITechGroup Inc., Logan, UT, USA). The intracellular buffer contained 90 mM CsF, 50 mM CsCl, 2.0 mM MgCl_2_·6 H_2_O, 10 mM NaF, 10 mM EGTA, and 10 mM HEPES (pH 7.4, adjusted with CsOH), and the osmotic pressure was adjusted to 261 mmol/kg using the same osmometer. Recordings were performed at room temperature (20–25 °C) using an EPC10 USB amplifier (HEKA Elektronik Dr. Schulze GmbH, Lambrecht, Pfalz, Germany) controlled by PatchMaster (HEKA Elektronik Dr. Schulze GmbH). The capillary resistance upon connecting the glass capillary, which was fabricated using a micropipette puller P-97 (Sutter Instruments, Novato, CA, USA), to the bath solution was 2–3 MΩ. Series resistance was 80% compensated. PST perfusion and Na_v_ functional observations were performed for as long as a resistance value of >1 GΩ was maintained.

The concentration-dependent inhibition of Na_v_ by the test compounds was investigated as follows. For the bivalve samples, serial dilutions of the extracts were prepared based on their chemical toxicity determined by LC-MS/MS. A series of test pulses— −100 mV (10 ms), −10 mV (20 ms), and −100 mV (10 ms)—was applied to the cells every 10 s. The cells were perfused with an extracellular buffer containing the test compound, and the inhibitory activity of the test compound was evaluated based on the decrease in peak current, while taking the run-down effect into account. The peak current was normalized and plotted against the concentration of the test compound and fitted to the Hill equation, Current = base + (max − base)/[1 + (IC_50_/Toxicity)^rate^], to construct a dose–response curve using Igor Pro 9 (Wave Metrics, Inc., Portland, OR, USA). The parameters base and max, representing the upper and lower asymptotes of the normalized current, respectively, were shared across the tested compounds, whereas the Hill coefficient (rate) and IC_50_ were fitted independently for each compound.

### 4.7. Statistical Analyses

Statistical analyses, statistical significance testing, and curve fitting were performed using Igor Pro 9 (Wave Metrics, Inc.). Data are presented as the mean ± standard deviation (SD) of independent experiments. The experimental groups consisted of independent experiments, and all statistical comparisons were non-directional. Normality and homogeneity of variance were assessed using the Shapiro–Wilk test [45] and the median-based Levene test [46], respectively. Differences among groups were analyzed using one-way ANOVA followed by Tukey’s multiple-comparison test [47]. A *p* value < 0.05 was considered statistically significant.

## Data Availability

The original contributions presented in this study are included in the article/Supplementary Materials. Further inquiries can be directed to the corresponding authors.

## Supplementary Materials

The following supporting information can be downloaded at https://www.mdpi.com/article/10.3390/md24100365/s1. Figure S1: Dose-dependent inhibition of endogenous Na_v_ in N2a cells by four toxic digestive glands from *Patinopecten yessoensis*; Table S1: SRM conditions of paralytic shellfish toxins; Table S2: Toxicity determined by the MBA and LC-MS/MS, and IC_50_ values obtained from 4 digestive glands of scallops *Patinopecten yessoensis* and those from whole body of *P. yessoensis*, *Mimachlamys nobilis*, *Perna viridis* and *Mytilus galloprovincialis*; Table S3: Global fitting of dose-dependence curves for paralytic shellfish toxin standards and bivalve extracts to the Hill’s Equation.

## Abbreviations

The following abbreviations are used in this manuscript:

PST	Paralytic shellfish toxin
STX	Saxitoxin
LC	Liquid chromatography
HPLC	High-performance liquid chromatography
MS	Mass spectrometry
LC-MS/MS	Liquid chromatography–Mass spectrometry/Mass spectrometry
MBA	Mouse bioassay
MU	Mouse Unit
CODEX	Codex Alimentarius Commission
FAO	Food and Agriculture Organization of the United Nations
WHO	World Health Organization
ELISA	Enzyme-Linked Immunosorbent Assay
TTX	Tetrodotoxin
Na_v_	Voltage-gated sodium channel
N2a	Neuroblastoma Neuro 2a
dc	decarbamoyl
dcSTX	Decarbamoyl STX
GTX	Gonyautoxin
EDTA	Ethylenediaminetetraacetic acid
HEPES	4-(2-Hydroxyethyl)-1-piperazineethanesulfonic acid
RT-PCR	Reverse transcription polymerase chain reaction
