# Supplementary material for: Electrophysiological Evaluation of Paralytic Shellfish Toxins in Bivalves
Source: Mar Drugs. 2026 Oct 19;24(10):365. doi: 10.3390/md24100365 (PMC13707111; doi:10.3390/md24100365)
Supplement: Supplementary file 1 [file marinedrugs-24-00365-s001.zip › marinedrugs-4568070-supplementary.pdf]

Supplementary Materials

Electrophysiological Evaluation of Paralytic Shellfish Toxins in Bivalves

Runa Yonekura [1,†], Satoshi Numano [2,†], Ryuichi Watanabe [2,*], Ikkei Yoshida [3,‡], Fumiko Obara [3], Junko Okumura [3,‡], Yuko Cho [1], Yuta Kudo [1,4], Kazuo Nagasawa [5], Mari Yotsu-Yamashita [1] and Keiichi Konoki [1,*]

[1] Graduate School of Agricultural Science, Tohoku University, 468-1 Aramaki-Aza-Aoba, Aoba-ku, Sendai 980-8572, Japan; yonekura.runa.r8@dc.tohoku.ac.jp (R.Y.); yuko.cho.a4@tohoku.ac.jp (Y.C.); yuta.kudo.d5@tohoku.ac.jp (Y.K.); mari.yamashita.c1@tohoku.ac.jp (M.Y.-Y.)

[2] Fisheries Technology Institute, Japan Fisheries Research and Education Agency, Yokohama, Kanagawa 236-8648, Japan; numano_satoshi55@fra.go.jp

[3] Japan Food Inspection Corporation, Sapporo Inspection Office, Rabo Bldg., Kita 1-jo Nishi 21-3-17, Chuo-ku, Sapporo, Hokkaido 064-0821, Japan; i_yoshida@jffic.or.jp (I.Y.); f_obara@jffic.or.jp (F.O.); j_okumura@jffic.or.jp (J.O.)

[4] Frontier Research Institute for Interdisciplinary Sciences, Tohoku University, 6-3 Aramaki-Aza-Aoba, Aoba-ku, Sendai 980-8578, Japan

[5] Department of Biotechnology and Life Science, Tokyo University of Agriculture and Technology, Koganei, Tokyo 184-8588, Japan; fx7239@go.tuat.ac.jp

[*] Correspondence: watanabe_ryuichi73@fra.go.jp (R.W.); keiichi.konoki.b2@tohoku.ac.jp (K.K.); Tel.: +81-22-757-4426 (K.K.)

[†] These authors contributed equally to this work.

[‡] Current Address: Japan Food Inspection Corporation, JS Progres Bldg. 3F, 4-1-23 Heiwajima, Ota-ku, Tokyo 143-0006, Japan.

Contents

Figure S1. Dose-dependent inhibition of endogenous Na_v in N2A cells by four toxic digestive glands from *Patinopecten yessoensis*.

Table S1. SRM conditions of paralytic shellfish toxins.

Table S2. Toxicity determined by the MBA and LC-MS/MS, and IC_{50} values obtained from 4 digestive glands of scallops *Patinopecten yessoensis* and those from whole body of *P. yessoensis*, *Mimachlamys nobilis*, *Perna viridis* and *Mytilus galloprovincialis*.

Table S3. Global fitting of dose-dependence curves for paralytic shellfish toxin standards and bivalve extracts to the Hill Equation

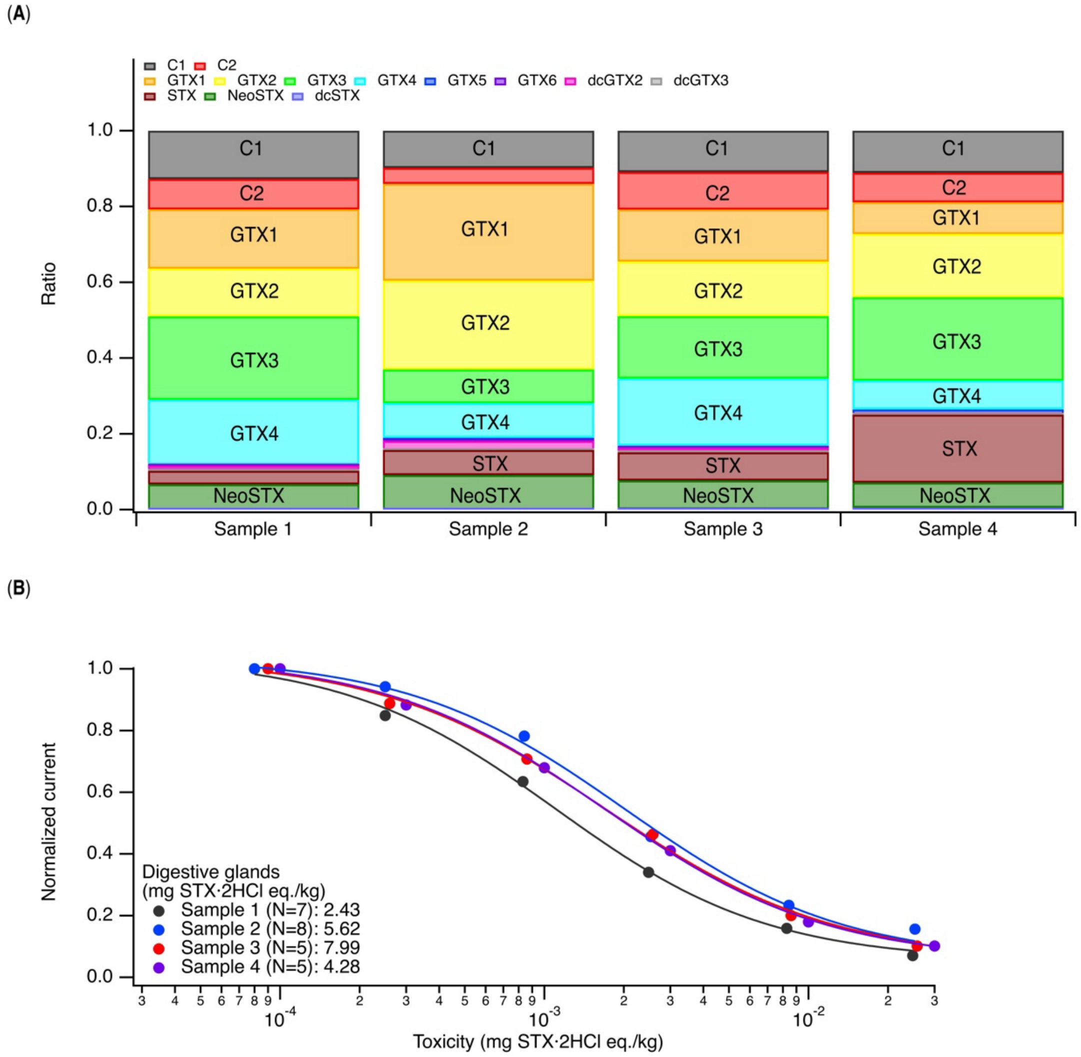

Figure S1. Dose-dependent inhibition of endogenous Na_v in N2A cells by four toxic digestive glands from *Patinopecten yessoensis*. (**A**) The PST composition of the four samples, Samples 1–4, was determined using LC-MS/MS. The total toxicity of Samples 1–4 was calculated to be 2.43, 5.62, 7.99, and 4.28 mg STX•2HCl eq./kg, respectively. The solution was diluted with extracellular buffer to the designated toxicity shown in the figure and then perfused into N2A cells. Plots of normalized sodium current versus toxicity were fitted to the Hill equation, and IC_{50} values were determined using the fitted curves. The IC_{50} value (μg STX•2HCl eq./kg) of Samples 1–4 was 1.14 ± 0.41 (N = 6), 1.86 ± 0.76 (N = 6), 2.10 ± 0.57 (N = 5), and 1.70 ± 0.21 (N = 5) (mean ± SD). One-way ANOVA revealed no significant difference among the four groups (P = 0.37).

Table S1. SRM conditions of paralytic shellfish toxins.

Analyte	Precursor ion (*m/z*)	Product ion (*m/z*)	DP (V)	CE (V)	CXP (V)	Ionization mode	Dwell time (msec)
STX	300.1	204.1 [(a)]<br>138.0 [(b)]	33<br>33	31<br>32	15<br>15	+<br>+	24.0<br>24.0
dcSTX	257.1	126.1 [(a)]<br>197.1 [(b)]	91<br>96	33<br>13	10<br>6	+<br>+	48.0<br>48.0
neoSTX	316.1	220.0 [(a)]<br>126.0 [(b)]	96<br>91	31<br>43	6<br>10	+<br>+	48.0<br>48.0
dcneoSTX	273.1	126.0 [(a)]<br>225.1 [(b)]	60<br>60	35<br>30	10<br>10	+<br>+	12.0<br>12.0
GTX1	410.1	367.1 [(a)]<br>349.1 [(b)]	- 190<br>- 190	- 24<br>- 32	- 7<br>- 19	−<br>−	12.0<br>12.0
GTX2	394.1	351.1 [(a)]<br>333.1 [(b)]	- 60<br>- 65	- 26<br>- 36	- 1<br>- 27	−<br>−	12.0<br>12.0
GTX3	394.1<br>396.1	333.1 [(a)]<br>298.1 [(b)]	- 65<br>51	- 36<br>23	- 27<br>22	−<br>+	12.0<br>12.0
GTX4	412.1<br>410.1	314.1 [(a)]<br>367.1 [(b)]	31<br>- 190	41<br>- 24	10<br>- 7	+<br>−	12.0<br>12.0
GTX5	380.1<br>378.1	300.1 [(a)]<br>122.0 [(b)]	31<br>- 135	19<br>- 28	20<br>- 14	+<br>−	12.0<br>12.0
GTX6	396.1<br>394.1	316.1 [(a)]<br>122.0 [(b)]	51<br>- 35	15<br>- 28	10<br>- 16	+<br>−	12.0<br>12.0
dcGTX2	351.1	164.0 [(a)]<br>333.1 [(b)]	- 170<br>- 170	- 38<br>- 24	- 16<br>- 10	−<br>−	12.0<br>12.0
dcGTX3	353.1<br>351.1	255.1 [(a)]<br>333.1 [(b)]	31<br>- 170	29<br>- 24	16<br>- 10	+<br>−	12.0<br>12.0
C1	474.1	122.0 [(a)]<br>351.1 [(b)]	- 60<br>- 60	- 36<br>- 30	- 21<br>- 7	−<br>−	12.0<br>12.0
C2	396.1<br>474.1	298.1 [(a)]<br>122.0 [(b)]	51<br>- 60	23<br>- 44	22<br>- 11	+<br>−	12.0<br>12.0
TTX	320.1	302.0 [(a)]<br>162.0 [(b)]	106<br>106	33<br>51	20<br>12	+<br>+	12.0<br>12.0

STX, Saxitoxin; GTX: Gonyautoxin; dc, decarbamoyl; TTX, Tetrodotoxin;

DP, Declustering potential; CE, collision energy; CXP, collision cell exit potential.

a) Used for quantitation, b) Used for confirmation

Table S2. Toxicity determined using the MBA and LC-MS/MS, and IC_{50} values (mean ± SD) obtained from four digestive glands of the scallop *Patinopecten yessoensis* and those from whole body of *P. yessoensis*, *Mimachlamys nobilis*, *Perna viridis,* and *Mytilus galloprovincialis*.

Species	Organ	MBA	LC-MS/MS	Electrophysiology	
		Toxicity		IC_{50}	N
		MU/g	mg STX・2HCl eq./kg	μg STX・2HCl eq./kg	
P. yessoensis	Digestive gland	12	2.43	1.14 ± 0.41	6
		37	5.62	1.86 ± 0.76	6
		70	7.99	2.10 ± 0.57	5
		41	4.28	1.70 ± 0.21	5
P. yessoensis	Whole body	2.6	0.35	1.06 ± 0.18	5
M. nobilis		11	2.19	1.74 ± 0.87	6
P. viridis		-	10.60	1.80 ± 0.46	6
M. galloprovincialis		26	4.41	1.76 ± 0.29	8
M. nobilis		-	0.07	1.25 ± 0.42	3
Reference standards	dcSTX			4.13 ± 0.45	3
	GTX2/3			1.41 ± 0.36	5
	C1/2			2.38 ± 0.87	5

Table S3. Global fitting of dose–response curves for PST reference standards and PST-contaminated shellfish extracts to the Hill equation*.

	IC_{50} (µg STX eq./kg)	*max*	*base*	*rate*
dcSTX	4.13 ± 0.45	0.06 ± 0.02	1.01 ± 0.01	1.03 ± 0.16
GTX2/3	1.41 ± 0.36	0.06 ± 0.02	1.01 ± 0.01	1.15 ± 0.22
C1/2	2.38 ± 0.87	0.06 ± 0.02	1.01 ± 0.01	0.87 ± 0.09
P. yessoensis	1.43 ± 0.02	0.08 ± 0.02	1.03 ± 0.01	1.24 ± 0.16
M. galloprovincialis	1.76 ± 0.29	0.08 ± 0.02	1.03 ± 0.01	1.19 ± 0.28
P. viridis	1.80 ± 0.45	0.08 ± 0.02	1.03 ± 0.01	1.10 ± 0.22
M. nobilis	1.25 ± 0.42	0.08 ± 0.02	1.03 ± 0.01	1.07 ± 0.01
M. nobilis	2.31 ± 1.06	0.08 ± 0.02	1.03 ± 0.01	1.14 ± 0.30

*The Hill equation is as follows, where *base*, *max*, IC_{50}, and *rate* are constants:

$$Current = base + \frac{max - base}{1 + \left(\frac{IC_{50}}{Toxicity}\right)^{rate}}$$

Toxicity and *Current* are variables representing the toxicity in the perfusion solution and the observed normalized current value, respectively. The experimental data were fitted to the Hill's equation using Igor Pro 9. The data are expressed as the mean ± standard deviation (SD). Based on the obtained constants, an equation was derived to calculate the toxicity of the test solution for perfusing N2A cells from the observed normalized current value, which is presented below.

$$Toxicity = IC_{50} \div \left(\frac{max - Current}{Current - base}\right)^{\frac{1}{rate}}$$
